# Development and validation of a stock addiction inventory (SAI)

**DOI:** 10.1186/s12991-016-0105-3

**Published:** 2016-07-28

**Authors:** HyunChul Youn, Jung-Seok Choi, Dai-Jin Kim, Sam-Wook Choi

**Affiliations:** 1 Chungmugong Leadership Center, Naval Education and Training Command, Republic of Korea Navy, 111, Jinhui-ro, Jinhae-gu, P.O. Box 211, Changwon-si, Gyeongsangnam-do 51655 South Korea; 2 Department of Psychiatry, SMG-SNU Boramae Medical Center, 20 Boramae-ro 5-gil, Dongjak-gu, Seoul, 07061 South Korea; 3 Department of Psychiatry, Seoul St Mary’s Hospital, College of Medicine, The Catholic University of Korea, 222 Banpo-daero, Seocho-gu, Seoul, 06591 South Korea; 4 Korea Institute on Behavioral Addictions, True Mind Clinic, F7, KR Tower, 1 141, Teheran-ro, Gangnam-gu, Seoul, 06132 South Korea; 5 Healthcare & Information Research Institute, Namseoul University, 91, Daehak-ro, Seonghwan-eup, Seobuk-gu, Cheonan-si, Chungcheongnam-do 31021 South Korea

**Keywords:** Stock addiction inventory, Financial markets, Stock investments, Trading, Gambling addiction, Behavioral addiction

## Abstract

**Background:**

Investing in financial markets is promoted and protected by the government as an essential economic activity, but can turn into a gambling addiction problem. Until now, few scales have widely been used to identify gambling addicts in financial markets. This study aimed to develop a self-rating scale to distinguish them. In addition, the reliability and validity of the stock addiction inventory (SAI) were demonstrated.

**Methods:**

A set of questionnaires, including the SAI, south oaks gambling screen (SOGS), and DSM-5 diagnostic criteria, for gambling disorder was completed by 1005 participants. Factor analysis, internal consistency testing, *t* tests, analysis of variance, and partial correlation analysis were conducted to verify the reliability and validity of SAI.

**Results:**

The factor analysis results showed the final SAI consisting of two factors and nine items. The internal consistency and concurrent validity of SAI were verified. The Cronbach’s *α* for the total scale was 0.892, and the SAI and its factors were significantly correlated with SOGS.

**Conclusions:**

This study developed a specific scale for financial market investments or trading; this scale proved to be reliable and valid. Our scale expands the understanding of gambling addiction in financial markets and provides a diagnostic reference.

## Background

Gambling addiction is characterized by recurrent and progressive maladaptive patterns of gambling behaviors, followed by a significant impairment in financial and psychosocial areas [1]. It was previously classified as one of the impulse control disorders, but since the publication of the fifth edition of the diagnostic and statistical manual of mental disorders (DSM-5), it has been included in the substance-related and addictive disorders [2–4]. This diagnostic change means that the gambling addiction now fulfills the diagnostic criteria for addiction, such as tolerance and craving [5].

In general, the prevalence of the gambling addiction is known as 1–2 % of the global population [5]. In South Korea, the National Gaming Control Commission and the Korea Center on Gambling Problems reported the prevalence of gambling addiction using the problem gambling severity index (PGSI) [6, 7]. According to the 2014 reports on the general population, 3.9 % of the respondents belonged to the moderate risk group and 1.5 % were problem gamblers.

These institutions mainly focused on playing casinos or lotteries, betting on sports or online, and betting on horse, bicycle, or motorboat racing [6, 7]. They reported that some financial market investors asked for help from the public services for gambling addiction, but whether investing in financial markets is gambling was described as “under-discussion.” We think that this is because financial market investments or trading is a normal economic activity. That is, financial market investments or trading is an economic activity for most investors, but can be gambling for some investors. The previous studies reported that financial market investments or trading can display the usual features of gambling [8, 9]. According to these studies, investing in financial markets always entails certain risks similar to gambling; gambling addicts in financial markets were characterized by high financial risks, lack of risk calculations, and high levels of sensation seeking. Another study regarded stock market gambling as one of the socially acceptable types of gambling and included it in the analyses along with the traditional gambling [10].

However, there have been few studies on gambling addiction in financial markets. Some researchers also reported that this is the least studied major area of gambling [11, 12]. According to the 2014 statistical data from the Korea Exchange, the investing population was estimated to be upwards of 5.07 million in South Korea [13]. This figure is 10.12 % of the total population and 19.72 % of the economically active population. Nevertheless, there are no statistical data or detailed research on how many people have gambling addiction problems in financial markets. To understand gambling addiction in financial markets and to study further, we believe that it is essential to distinguish gambling addicts in financial markets. Until now, few scales have widely been used to identify gambling addicts in financial markets.

This study aimed to develop a self-rating scale to distinguish gambling addicts in financial markets. Initially, we developed the first version of the stock addiction inventory (SAI). Then, after revising the scale via factor analysis, the final SAI was completed. In addition, the reliability and validity of the SAI were demonstrated.

## Methods

### Participants and procedure

A total of 1005 adults (over 20 years of age) who had engaged in financial market investments or trading within 1 month from the start of the study participated (data collected from September 22–October 4, 2014). Korea’s most prestigious market and opinion research firm (KMPMORF) completed data collection. KMPMORF is a research company with about 1.19 million panels recruited randomly in South Korea [14]. Our participants were recruited randomly among these panels. 1500 KRW (approx. 1.3 USD) was offered to each participant that fully completed the survey. Written informed consent was obtained from participants prior to their survey inclusion. The institutional review board at Eulji University Hospital (reference 12-068) provided the necessary ethical permissions to conduct the study. We used the computer-aided Web interview method, so we could exclude participants with missing data prior to the final data collection and analysis.

### Measures

All questionnaires were in the self-report format. The beginning of the first questionnaire contained items assessing the following demographic characteristics: age, gender, years of education, marital status, and monthly income.

We referred to existing scales for “gambling” to increase the content validity in the SAI development process, because the SAI focused on “gambling” addiction in financial markets. The first SAI was developed via modifications of the PGSI, financial markets gambling questionnaire (FMGQ), and DSM-5 diagnostic criteria for gambling disorder. The PGSI contains nine items with a four-point response format ranging from “never” (zero) to “almost always” (three) [15]; it has widely been used to screen for gambling addiction in the general population. The FMGQ was developed to screen problem gambling in financial markets by the Connecticut Council on Problem Gambling and contains 20 items [16]. We adopted these items to include more specific questions for financial market gambling. In addition, we added items of the DSM-5 diagnostic criteria for gambling disorder to the first SAI to better screen disordered gamblers. We also created one item that represented “craving” by reference to the DSM-5 diagnostic criteria for other substance use disorders. In addition, each of the items was revised appropriately for financial market gambling. For example, we changed the term “gambling” to “investments or trading.” Finally, the first 21-item SAI was completed (Table 1). We adopted a four-point response format ranging from “never” (zero) to “almost always” (three) like the PGSI instead of “yes” and “no” like the FMGQ and DSM-5 diagnostic criteria for gambling disorder; this facilitated the participants’ responses, as it makes the decision easier and more realistic. We mentioned “within the last 12 months” in the “instructions” of the SAI as in the PGSI, FMGQ, and DSM-5 diagnostic criteria for gambling disorder. Five gambling addiction field professionals who were bilingual in Korean and English (two psychiatrists and three psychologists) conducted these processes and revisions.

**Table 1:** First version of SAI

No.	Question	Major reference (minor references)
1	Having borrowed money from friends or financial institutions or sold anything to get money to invest or trade	PGSI (FMGQ, DSM-5 gambling disorder)
2	Having regretted or felt guilty about my excessive investments or trading	PGSI
3	Having heard from others that I had problems with my investments or trading, regardless of whether I thought it was true	PGSI (FMGQ)
4	My investments or trading has caused financial problems for me or my household	PGSI
5	Having bet more than I could really afford to lose	PGSI (FMGQ)
6	Having returned another day to try to win back the money I lost when investing or trading	PGSI (FMGQ, DSM-5 gambling disorder)
7	My investments or trading has caused me health problems, including stress or anxiety	PGSI
8	Having invested or traded to escape or alleviate negative moods (e.g., depression, anxiety, helplessness, guilt, stress)	DSM-5 gambling disorder (FMGQ)
9	Having lied to family or others about how much I invest or trade or the amount of money involved in my investments or trading	DSM-5 gambling disorder (FMGQ)
10	Having failed in my attempt to cut down or stop my investments or trading	DSM-5 gambling disorder (FMGQ)
11	Having neglected or felt difficulties in family, occupational, or social lives, because of my investments or trading	DSM-5 gambling disorder (FMGQ)
12	Having felt a craving or a strong desire or urge to make a great deal of money from my investments or trading	DSM-5 other substance use disorders
13	Having spent increasing time or money on my investments or trading	PGSI (FMGQ, DSM-5 gambling disorder)
14	Having been nervous, irritable, or anxious when trying to cut down or stop my investments or trading	DSM-5 gambling disorder
15	Thoughts of reliving past investing or trading experiences, or expectations for next investments or trading have been an important part of my daily life	DSM-5 gambling disorder
16	Having been preoccupied with the status of my investments or trading and have frequently checked on whether returns have gone up or down	FMGQ
17	My investments or trading has become increasingly speculative or risky over time	FMGQ
18	Having been restless or irritable when unable to be active in the markets, for example, when short of money, away on vacation, trying to cut back on my trading	FMGQ
19	Having felt uncomfortable when any cash accumulated in my brokerage account and have tried to quickly find a way to keep it in action	FMGQ
20	Having not opened brokerage statements to avoid having to think about my losses	FMGQ
21	Having felt excessive expectation or suspense when checking my stock prices	FMGQ

*SAI* stock addiction inventory, *PGSI* problem gambling severity index, *FMGQ* financial markets gambling questionnaire, *DSM*-*5* diagnostic and statistical manual of mental disorders, fifth edition

To verify the concurrent validity of the SAI, the South Oaks Gambling Screen (SOGS) was added to the questionnaire. The SOGS is a 20-item self-report screening tool for gambling-related problems [17]. We adopted the SOGS, because the SAI focused on “gambling” addiction in financial markets. The SOGS discriminates “no problem” (total score = 0), “some problem” (total score between one and four), and “probable pathological gambler” (total score ≥ 5). The Korean version of the SOGS was developed in 2001 [18]; our study used this version. In this study, the internal consistency test result (Cronbach’s *α*) for the SOGS was 0.857.

The DSM-5 diagnostic criteria for gambling disorder consist of nine items [3] and were added to assess if the SAI can properly distinguish gambling addicts. We created nine self-report items based on these criteria by changing the term “gambling” to “investments or trading”; the cutoff was the answer “yes” to more than four questions as with the original criteria. In this study, the internal consistency test result (Cronbach’s *α*) for the DSM-5 diagnostic criteria for gambling disorder was 0.889.

### Statistical analysis

Principal axis factoring with direct oblimin rotation was conducted to determine the factor structure underlying the SAI items. We used the direct oblimin rotation, because the factors were considered to have relationships. The communalities and factor loads that were less than 0.4 were ignored. To estimate the reliability of the SAI, the internal consistency of the scale and factors was measured. We used the partial correlation analysis controlling age, gender, years of education, marital status, and monthly income to determine the concurrent validity. We also compared the SAI scores of non-addicts and addicts grouped by SOGS and DSM-5 diagnostic criteria for gambling disorder using independent *t* test (for two-group comparison) and analysis of variance (ANOVA, for three-group comparison).

All statistical tests were two sided. A *P* value of less than 0.05 was considered to indicate statistical significance. All statistical analyses were performed using the Statistical Package for Social Sciences version 18.0 for Windows (SPSS, Chicago, IL, USA).

## Results

Table 2 shows the general characteristics of the study participants.

**Table 2:** General characteristics of participants (*N* = 1005)

Characteristics	Categories	*N* (%) or mean ± SD
Age (year)		42.39 ± 10.16
Gender	Male	700 (69.7)
	Female	305 (30.3)
Education (year)		16.58 ± 8.28
Marital status	Unmarried	207 (20.6)
	Married (living with spouse)	765 (76.1)
	Divorce/separation	25 (2.5)
	Separation by death	8 (0.8)
Monthly income (million won)	<100	13 (1.3)
	100–199	41 (4.1)
	200–299	103 (10.2)
	300–399	200 (19.9)
	400–499	196 (19.5)
	500–599	164 (16.3)
	600–699	103 (10.2)
	≥700	185 (18.4)

### Factor structure (construct validity)

The Kaiser–Meyer–Olkin test of appropriateness of the sample was 0.970 and the *P* value of the Bartlett test was 0.000, which allowed for a pertinent factor analysis. We extracted two factors based on scree plot and principal axis factoring. Twelve items of the first SAI were excluded in the analysis process. The first SAI items 19 and 20 were excluded, because their communalities were less than 0.4. Items 6, 9, 10, 14, 17, and 18 were also excluded, because their factor loads were high on two factors. At this point, factor one consisted of items 1–5, 7, 8, and 11 and factor two consisted of items 12, 13, 15, 16, and 21. Item 8 represented “motive for investments or trading,” while the other items in factor one indicated problem gambling behavior and its consequences. That is, its meaning did not agree with those of other items in factor one. Item 11 represented “functional impairment” and was expected to associate with items 12, 13, and 15, which represented “craving,” “tolerance,” and “preoccupation,” respectively; these four items have been regarded as key characteristics of addictive disorders in various studies [3, 4, 19]. Item 11 was included in factor one, unlike items 12, 13, and 15. This might be because item 11 could also indicate the consequences of gambling. Therefore, we thought that item 11 had meanings that could be included in both factors. Items 16 and 21 were related only to FMGQ and added to include more specific questions for financial market gambling. However, all other items only related to the FMGQ were excluded in the factor analysis process. Items 16 and 21 were included in factor two, but their meanings did not agree with other items in factor two. For these reasons, we additionally excluded items 8, 11, 16, and 21.

In conclusion, the final SAI consisted of two factors and nine items (Table 3). Factor one grouped items one-to-six and their factor loads were 0.574–0.930. Factor two grouped items seven-to-nine and their factor loads were 0.652–0.805. These two factors explained 65.28 % of the entire scale.

**Table 3:** Factor analysis of SAI

No.	Question	Factor 1 (features of problem gambling)	Factor 2 (core features of addictive disorder)
1	Having borrowed money from friends or financial institutions or sold anything to get money to invest or trade	0.651	
2	Having regretted or felt guilty about my excessive investments or trading	0.668	
3	Having heard from others that I had problems with my investments or trading, regardless of whether I thought it was true	0.711	
4	My investments or trading has caused financial problems for me or my household	0.930	
5	Having bet more than I could really afford to lose	0.768	
6	My investments or trading has caused me health problems, including stress or anxiety	0.574	
7	Having felt a craving or a strong desire or urge to make a great deal of money from my investments or trading		0.652
8	Having spent increasing time or money on my investments or trading		0.741
9	Thoughts of reliving past investing or trading experiences, or expectations for next investments or trading have been an important part of my daily life		0.805
Eigenvalue		4.897	0.978
Variance (%)		54.407	10.870

*SAI* stock addiction inventory

### Internal consistency (reliability)

We calculated Cronbach’s *α* to estimate internal consistency. The Cronbach’s *α* for the total scale was 0.892 and those for two factors were 0.877 and 0.790, respectively (Table 4). These results permit the assertion that the items are homogenous and that the scale consistently measures the characteristics for which it was created.

**Table 4:** Reliability coefficients for SAI factors

Factor name	Mean ± SD	Cronbach’s α	Question	Corrected item-total correlations	Cronbach’s α if the item is eliminated
Features of problem gambling	2.24 ± 2.97	0.877	Q1	0.625	0.865
			Q2	0.680	0.857
			Q3	0.666	0.859
			Q4	0.772	0.841
			Q5	0.696	0.854
			Q6	0.664	0.859
Core features of addictive disorder	1.98 ± 1.80	0.790	Q7	0.592	0.760
			Q8	0.666	0.678
			Q9	0.639	0.708

*SAI* stock addiction inventory

### Correlations between SAI factors and SOGS (concurrent validity)

The results of the partial correlation analysis controlling age, gender, years of education, marital status, and monthly income show that all of the factors and total scores of SAI were significantly related to SOGS scores. Correlation coefficients for factor one, factor two, and total scores of SAI were 0.712, 0.638, and 0.752, respectively (all *P* < 0.01).

### SOGS, DSM-5, and SAI scores

To additionally confirm that SAI can properly distinguish gambling addicts, we compared the SAI scores of non-addicts and addicts grouped by SOGS and DSM-5 diagnostic criteria for gambling disorder. The results of independent *t* test (for DSM-5 diagnostic criteria for gambling disorder) and ANOVA (for SOGS) are shown in Table 5. In both analyses, the groups that had more gambling addiction problems showed significantly higher scores of SAI and its factors.

**Table 5:** Differences in SAI scores related to groups categorized by SOGS and DSM-5 diagnostic criteria scores

Variable	*N* (%)	SAI total		Factor 1		Factor 2	
		Mean ± SD	*P*	Mean ± SD	*P*	Mean ± SD	*P*
SOGS^a^			<0.001		<0.001		<0.001
=0	126 (12.5)	0.89 ± 2.14		0.37 ± 1.81		0.52 ± 0.81	
1–4	465 (46.3)	2.36 ± 2.46		1.01 ± 1.70		1.34 ± 1.33	
≥5	414 (41.2)	7.34 ± 4.55		4.19 ± 3.25		3.14 ± 1.80	
DSM-5^b^			<0.001		<0.001		<0.001
0–3	788 (78.4)	2.98 ± 3.10		1.45 ± 2.12		1.53 ± 1.46	
≥4	217 (21.6)	8.74 ± 5.18		5.12 ± 3.73		3.62 ± 1.98	

*SAI* stock addiction inventory, *SOGS* South oaks gambling screen, *DSM*-*5* diagnostic and statistical manual of mental disorders, fifth edition

^a^Analysis of variance (ANOVA)

^b^Independent *t* test

## Discussion

*This study developed the SAI.* All of the analyses described above indicate that the SAI was proven to distinguish gambling addicts in financial markets with high reliability and validity. The final SAI consisted of two factors and nine items.

Factor one included six items, which represented “borrowing money,” “guilt feelings,” “criticized by others,” “financial problems,” “betting more than can afford,” and “health problems,” respectively. These items were based on the PGSI and commonly indicated problem gambling behavior and its consequences [15]. Thus, we named factor one “features of problem gambling.”

Factor two consisted of three items, which represented “craving,” “tolerance,” and “preoccupation,” respectively. These were not only included in DSM-5 diagnostic criteria for other addictive disorders, such as substance use disorders, but also illustrated in various studies as key characteristics of addictive disorders [3, 4, 19]. Thus, we named factor two “core features of addictive disorder.”

We focused on characteristics of financial market investments or trading, which are different from other gambling, in the SAI development process. Unlike other gambling, investing in financial markets is promoted and protected by the government as an essential economic activity [9]. Stock market investors are usually regarded as persons with expertise, solid economic knowledge, and capable of understanding how a complex and organized structure, such as the stock market, works [8]. Because of these positive social evaluations, stock market gamblers may be considered better adapted than other gamblers, so the symptoms of gambling addiction can be easily overlooked, leading to underdiagnosis [8]. In addition, people begin to gamble initially out of curiosity or for fun, but people who invest in financial markets expect monetary rewards [9]. That is, most stock market investors mainly intend to make money and so do gambling addicts in financial markets.

These two features of financial market investors—social acceptance and monetary purposes—can affect the response tendencies to gambling scales, such as PGSI. Social acceptance can prevent investors’ insight into the fact that their investments or trading can be gambling. Thus, we thought that investors may be more likely to answer “no” to usual questions about gambling. This is one of the reasons; specific scales for financial market investors are required. In addition, the nuances of several questions may be changed by social acceptance. For example, borrowing money and chasing losses are generally negative behaviors in usual gambling, but they may be acceptable behaviors in the financial markets depending on the situation. In addition, we thought that monetary purposes would influence answers to depend on the monetary results of one’s investments or trading. For example, the gambling addicts who accidently earned a lot of money on financial markets may not feel guilty, not have insight, not be criticized by others, not have health or financial problems, and not borrow money. The cases of normal investors who unfortunately lose money on financial markets may be opposite. For clinicians, it is more important to distinguish gambling addicts, not the failed investors; thus, we thought that such problems caused by monetary purposes needed to be addressed.

Considering the above-mentioned features and problems, we developed a specific scale for financial market investments or trading. The PGSI that is the basis of the SAI consists of questions about “problem gambling behaviors” and “adverse consequences of gambling” [15]. We thought that the PGSI was not free of the problems described above; thus, we additionally used the FMGQ and DSM-5 diagnostic criteria for gambling disorder. The items only related to the FMGQ were all excluded in the analysis process. As a result, the final SAI consisted of seven items modified from the PGSI (No. 1–6, 8) and two additional items (No. 7, 9) representing craving and preoccupation, respectively. These two items are focused more on the pathologic aspects of addiction [4, 19] and help address the problems caused by specific features of financial market investments or trading as mentioned above. Furthermore, factor two named “core features of addictive disorder” includes three items (No. 7–9) that represent craving, tolerance, and preoccupation, respectively. We think that “core features of addictive disorder” can play an especially major role to distinguish gambling addicts from normal or failed investors in financial markets.

There are several limitations to this study. First, as the literature in this field is insufficient, the theoretical base of this study was relatively weak. Second, the questionnaires used in this study were self-administered, so we cannot rule out effects of denial or minimization by the respondents. Future study may benefit from combined use of questionnaires by spouses or parents. Third, our study did not investigate the diagnostic utility of the SAI. We think that cut-off scores between non-problem investors and gambling addicts in financial markets and comparisons with clinical interviews for financial market gambling addiction will be necessary. Fourth, confirmatory factor analysis was not conducted in this study and discriminant validity was not investigated. These analyses will help to confirm our findings.

## Conclusions

The implications drawn from the results of this study expand the understanding of gambling addiction in financial markets and provide a diagnostic reference. The SAI consists of nine questions and is grouped into two factors, all weighted equally on a four-point scale. The two factors’ scores are summed to yield a total SAI score with a 0–27 range, where a higher score indicates a higher possibility of having a gambling addiction problem in financial markets.

Gambling addiction in financial markets is an unexplored issue, so there are very few studies on it. We think that it is necessary to collect more clinical data about financial market gambling and to increasingly study the clinical features of this gambling, such as depression, anxiety, and impulses. Our study is just a beginning for the clinical diagnosis of financial market gambling, so such further studies will be able to verify and advance the results of our study.

## Abbreviations

SAI: stock addiction inventory
SOGS: south oaks gambling screen
DSM-5: the fifth edition of the diagnostic and statistical manual of mental disorders
PGSI: problem gambling severity index
KMPMORF: Korea’s most prestigious market and opinion research firm
FMGQ: financial markets gambling questionnaire
ANOVA: analysis of variance
SPSS: statistical package for social sciences
